# Hepatitis C diagnosics: clinical evaluation of the HCV-core antigen determination

**DOI:** 10.1186/1471-2334-14-S2-P88

**Published:** 2014-05-23

**Authors:** Josef van Helden

**Affiliations:** 1 MVZ Dr. Stein und Kollegen, Moenchengladbach, Germany

## Background

The aim of the evaluation was to investigate the relevance of the HCV-core antigen testing for the diagnosis and monitoring of HCV infections in the daily routine. Up to now, most of the serological diagnostics was performed as determination of antibodies while the determination of activity and the monitoring of antiviral therapy were checked by HCV RNA PCR.

## Methods

The routine requests for HCV-core antigen of a private laboratory were analyzed for a period of two years.

## Results

The determination of HCV antigen highly correlates with the quantitative measurement of HCV RNA (r=0.73), p=0.0003). The diagnostic window is comparable with that of the HCV PCR (27.1 ± 12.8 d vs. 23.9 ± 9.2 d, p=0.11). The sensitivity of the HCV antigen assay was 99.0 % with a specificity of 99.2 %. 54.3 % of the confirmed antibody positive samples were also antigen positive. Only in 3 of 560 HCV-RNA positive samples HCV antigen was not detectable, but 3 samples without HCV antibodies were confirmed positive for HCV antigen.

## Conclusions

The HCV antigen assay is a suitable tool for the detection of chronic active HCV infections, for the early diagnosis of acute infections and for testing of HCV in patient with immunodeficiency. The HCV antigen assay valuable completion of serological testing for HCV.

